# HmsB enhances biofilm formation in *Yersinia pestis*

**DOI:** 10.3389/fmicb.2014.00685

**Published:** 2014-12-12

**Authors:** Nan Fang, Shi Qu, Huiying Yang, Haihong Fang, Lei Liu, Yiquan Zhang, Li Wang, Yanping Han, Dongsheng Zhou, Ruifu Yang

**Affiliations:** State Key Laboratory of Pathogen and Biosecurity, Beijing Institute of Microbiology and EpidemiologyBeijing, China

**Keywords:** *Yersinia pestis*, HmsB, c-di-GMP, biofilm

## Abstract

The *hmsHFRS* operon is responsible for biosynthesis and translocation of biofilm matrix exopolysaccharide. *Yersinia pestis* expresses the two sole diguanylate cyclases HmsT and HmsD and the sole phosphodiesterase HmsP, which are specific for biosynthesis and degradation, respectively, of 3′,5′-cyclic diguanosine monophosphate (c-di-GMP), a second messenger promoting exopolysaccharide production. In this work, the phenotypic assays indicates that *Y. pestis* sRNA HmsB enhances the production of c-di-GMP, exopolysaccharide, and biofilm. Further gene regulation experiments disclose that HmsB stimulates the expression of *hmsB*, *hmsCDE*, *hmsT*, and *hmsHFRS* but represses that of *hmsP*. HmsB most likely acts as a major activator of biofilm formation in *Y. pestis*. This is the first report of regulation of *Yersinia* biofilm formation by a sRNA. Data presented here will promote us to gain a deeper understanding of the complex regulatory circuits controlling *Yersinia* biofilm formation.

## Introduction

*Yersinia pestis* is the causative agent of plague, one of the most dangerous infectious diseases. Flea-borne transmission of *Y. pestis* occurs among mammals including humans, which distinguishes this pathogen from its genetically close progenitor *Y. pseudotuberculosis* that is a mild food-borne enteric pathogen (Zhou and Yang, 2011). *Y. pestis* biofilms, a population of bacterial colonies embedded in a self-produced exopolysaccharide matrix (Darby, 2008; Hinnebusch and Erickson, 2008; Zhou and Yang, 2011), can attach to and physically block flea's proventriculus. The inability to take in a blood meal when the proventriculus is blocked makes fleas feel hungry and bite repeatedly and thereby promoting *Y. pestis* to be spread into new individuals of mammalian reservoirs (Darby, 2008; Hinnebusch and Erickson, 2008; Zhou and Yang, 2011).

*Yersinia pestis* biofilms can also block feeding of model nematode *Caenorhabditis elegans*, because attached biofilms are primarily found on the larva head to blanket the mouth (Darby et al., 2002; Fang et al., 2013). By contrast, most strains of *Y. pseudotuberculosis* have the biofilm-negative phenotype, although a few of them (being similar to *Y. pestis*) can form robust biofilms at gas-liquid-solid interfaces or on nematodes (Erickson et al., 2006; Fang et al., 2013).

*hmsHFRS*, *hmsCDE*, *hmsT*, and *hmsP* encode the major factors involved in biofilm formation of *Y. pestis*. HmsHFRS are responsible for biosynthesis and translocation of exopolysaccharide through cell envelope (Bobrov et al., 2008; Hinnebusch and Erickson, 2008), HmsT and HmsD are the two sole diguanylate cyclases, which are responsible for biosynthesis of 3′,5′-cyclic diguanosine monophosphate (c-di-GMP), a second messenger promoting exopolysaccharide production (Bobrov et al., 2011; Sun et al., 2011). HmsP is the sole phosphodiesterase responsible for c-di-GMP degradation (Kirillina et al., 2004; Bobrov et al., 2005).

cDNA cloning approach and deep sequencing technology have been used for global identification of small RNA (sRNA) candidates in *Y. pestis* (Qu et al., 2012; Beauregard et al., 2013; Yan et al., 2013; Schiano et al., 2014) and *Y. pseudotuberculosis* (Koo et al., 2011). However, only three specific sRNA, namely Ysr141 (Schiano et al., 2014), GcvB (McArthur et al., 2006), and RyhB (Deng et al., 2014), have been characterized for their contribution to gene regulation in *Y. pestis*.

**Figure 1:**
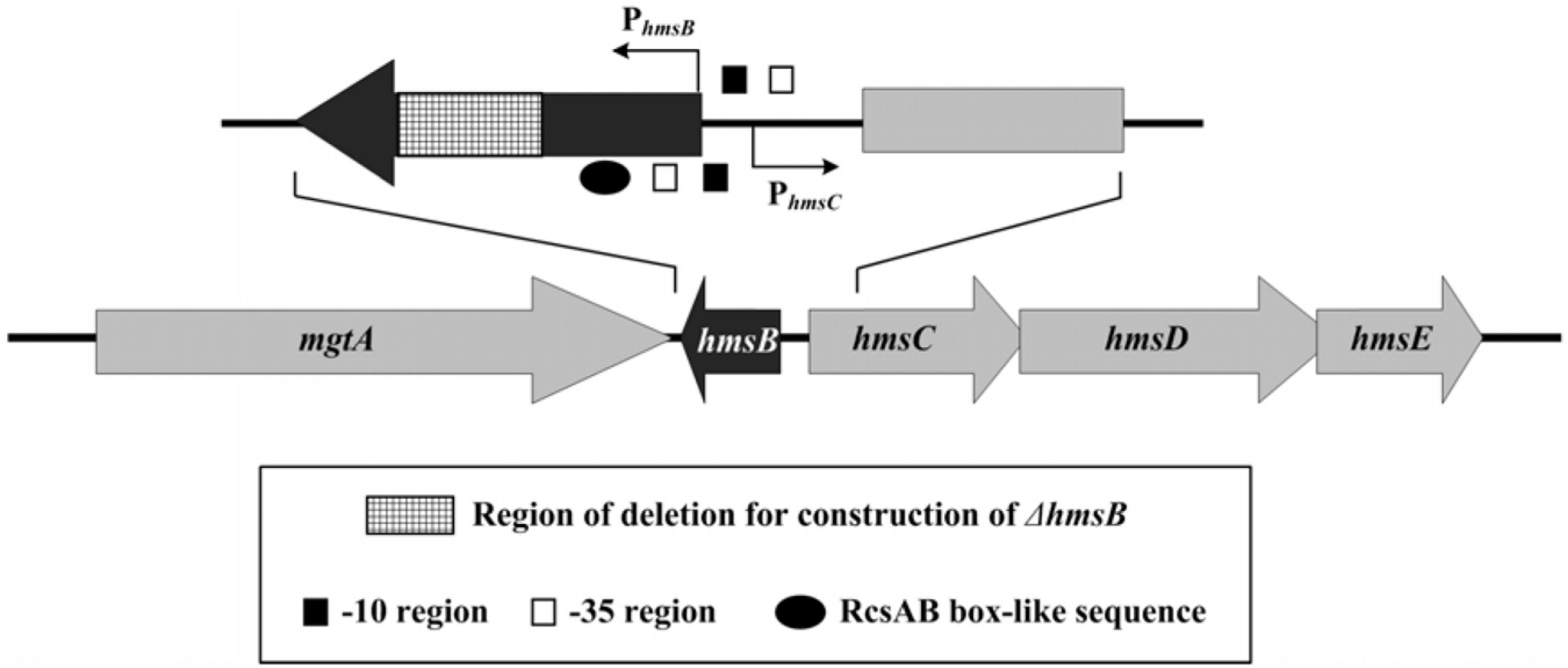
**Genetic organization of *hmsB* and *hmsCDE***. Boxed arrows represented length and direction of indicated ORFs. Broken arrows indicated transcription starts (i.e., transcribed promoters). Please refer to our companion submission for regulatory action of RcsAB on *hmsB* and *hmsCDE*.

Our previous RNA-seq study of *Y. pestis* (Yan et al., 2013) identified a temperature-dependent sRNA named sRNA035 located nearby *hmsCDE* (Figure 1), which promoted us to test whether sRNA035 was involved in regulating the production of c-di-GMP and biofilm formation. This sRNA was designated HmsB herein. This follow-up study disclosed that HmsB positively regulated *hmsCDE*, *hmsT*, *hmsHFRS* and its own gene but negatively regulated *hmsP*, and thus acted as a major activator of c-di-GMP, exopolysaccharide and biofilm production in *Y. pestis*.

## Experimental procedure

### Bacterial strains

The wild-type *Yersinia pestis Microtus* strain 201 (WT) is avirulent to humans but highly virulent to mice (Zhou et al., 2004). The partial coding region of each indicated gene was replaced by the kanamycin resistance cassette by using the one-step inactivation method based on the lambda phage recombination system (Datsenko and Wanner, 2000), to generate the corresponding mutant of *Y. pestis* (Table 1). For *in trans* complementation, a PCR-generated DNA fragment containing the coding region of each indicated gene together with its promoter-proximal region and transcriptional terminator-proximal region was cloned into the cloning vector pBluescript II KS(+) (Agilent Technologies). The resulting recombinant vector was transformed into indicated *Y. pestis* strain lack of the corresponding functional gene, generating the corresponding complemented mutant (Table 1). All the primers designed in this study were listed in Table S1.

**Table 1:** ***Y. pestis* strains involved in gene deletion and complementation**.

**Strain**	**Functional (+) or inactivated (−)**						**Feature**	**References**
	***hmsB***	***hmsD***	***hmsT***	***hmF***	***hmsS***	***hmsP***		
WT	+	+	+	+	+	+	The wild-type *Y. pestis* biovar *Microtus* strain 201.	Zhou et al., 2004
							The *rscA* gene was inactivated naturally.	
ΔhmsB	−	+	+	+	+	+	The base pairs 103 to 229 of *hmsB* gene was deleted from WT.	This study
*c-hmsB*	+	+	+	+	+	+	The vector pBluescript-*hmsB* was introduced into ΔhmsB.	This study
ΔhmsD	+	−	+	+	+	+	The base pairs 41 to 1238 of *hmsD* gene was deleted from WT.	This study
Δ*hmsT*	+	+	−	+	+	+	The base pairs -4 to 1179 of *hmsT* gene was deleted from WT.	This study
ΔhmsT ΔhmsD	+	−	−	+	+	+	A reference c-di-GMP-negative strain.	This study
							The base pairs 41 to 1238 of *hmsD* gene was deleted from Δ*hmsT*.	
Δ*hmsF*	+	+	+	−	+	+	The base pairs 216 to 1764 of *hmsF* gene was deleted from WT.	This study
ΔhmsS	+	+	+	+	−	+	A reference biofilm-negative strain.	Sun et al., 2012
							The base pairs 146 to 468 of *hmsS* was deleted from WT.	
Δ*hmsP*	+	+	+	+	+	−	The base pairs 172 to 2187 of *hmsP* gene was deleted from WT.	This study

### Bacterial growth and RNA isolation

Overnight cell cultures in the Luria-Bertani (LB) broth with an optical density (OD_620_) of about 1.0 were diluted 1:50 into 18 ml of fresh LB broth for further cultivation at 26°C with shaking at 230 rpm to reach the middle stationary phases (an OD_620_ of 0.8–1.2), followed by cell harvest for further gene regulation or phenotypic assays. Immediately before bacterial harvest for RNA isolation, double-volume of RNAprotect reagent (Qiagen) was added to one-volume of cell culture, and total RNA was extracted using TRIzol Reagent (Invitrogen). RNA quality was monitored by agarose gel electrophoresis, and RNA quantity was determined by spectrophotometry.

### 5′-race and 3′-race

Following generation of cDNA sample from total RNA through reverse transcription (RT), 5′- or 3′-rapid amplification of cDNA ends was done using SMARTer RACE cDNA Amplification Kit. After agarose gel electrophoresis, the 5′-RACE or 3′-RACE fragment was recovered and purified with TaKaRa MiniBEST Agarose Gel DNA Extraction Kit, and sequenced with ABI-3700 automated DNA sequencer.

## Primer extension assay

As described in our previous studies (Sun et al., 2012; Zhang et al., 2013a,b), a 5′−^32^P-labeled oligonucleotide primer complementary to a portion of the RNA transcript of each indicated gene was employed to synthesize cDNAs from total RNA templates using Promega Primer Extension System. If different *Y. pestis* strains were involved in a single experiment, equal amounts of total RNA were used as starting materials. Sequence ladders were prepared with the same 5′−^32^P-labeled primers using AccuPower & Top DNA Sequencing Kit (Bioneer). Radioactive species were detected by autoradiography. The 5′-terminus of RNA transcript (i.e., transcription start) of each target gene was mapped according to size of primer extension product, while the relative mRNA levels were determined with intensities of primer extension product.

### LacZ fusion and β galactosidase assay

A promoter-proximal DNA region of each indicated gene was cloned into the low-copy-number transcriptional fusion vector pRW50 (Lodge et al., 1992) that harbors a promoterless *lacZ* reporter gene. *Y. pestis* strains transformed with the recombinant plasmid or the empty pRW50 (negative control) were grown to measure β-galactosidase activity in cellular extract using β-Galactosidase Enzyme Assay System (Promega) (Sun et al., 2012; Zhang et al., 2013a,b).

### Antibody preparation and western blot

The 6× His-tagged peptide fragments of HmsT (a.a.285–390), HmsD (a.a.221–425), HmsF (a.a.193–482), and HmsP (a.a.441–671) were over-expressed, respectively, in BL21 (DE3) cells using pET28a vectors. Each recombinant protein was purified under denaturing conditions with Ni-NTA Agarose Column, and further prepared as soluble protein sample after renaturation for further immunization of New Zealand rabbits. The specific polyclonal IgG antibody was separated from rabbit serum by ammonium sulfate precipitation. For Western blot, cleared whole-cell lysate was prepared from harvested bacterial cells through sonication, followed by determination of protein concentrations with Bio-Rad protein assay kit. If different *Y. pestis* strains were involved in a single experiment, equal amounts of protein sample were separated on SDS-PAGE, immunoblotted to polyvinylidene fluoride membranes (Immobilon P; Millipore), and incubated with primary antibody and then goat anti-rabbit IRDye®800CW second antibody. Signals were detected with Odyssey Sa Infrared Imaging System.

### Biofilm and c-di-GMP assays

As described in our previous study (Fang et al., 2013), three different methods were used to detect *Y. pestis* biofilms. First, *in vitro* biofilm masses, attached to well walls when bacteria were grown in polystyrene microtiter plates, were stained with crystal violet. Second, percentages of fourth-stage larvae and adults (L4/adult) of *C. elegans* after incubation of nematode eggs on *Y. pestis* lawns, negatively reflecting bacterial ability to produce biofilms, were determined. Third, rugose colony morphology of bacteria grown on LB agar plates, positively reflecting bacterial ability to synthesize exopolysaccharide, was observed. In addition, intracellular c-di-GMP levels were determined by a chromatography-coupled tandem mass spectrometry (HPLC-MS/MS) method as described in our previous study (Sun et al., 2012).

### Experimental replicates and statistical methods

For LacZ fusion, crystal violet staining of biofilms, and determination of L4/adult nematodes or c-di-GMP, experiments were performed with at least three independent bacterial cultures/lawns, and values were expressed as mean ± standard deviation. Paired Student's *t*-test was performed to determine statistically significant differences; *P* < 0.01 was considered to indicate statistical significance. For primer extension, Western blot, and colony morphology observation, representative data from at least two independent bacterial cultures were shown.

## Results

The 5′ and 3′ termini of HmsB were determined by 5′-RACE and 3′-RACE, respectively. The 262-bp *hmsB* gene, situated from nucleotide position 4,72,430–4,72,691 on *Y. pestis* CO92 genome, was located within the intergenic region of *mgtA* (YPO0451) and *hmsC*, and the two adjacent genes *hmsB* and *hmsC* were transcribed with opposite direction (Figure 1).

**Figure 2:**
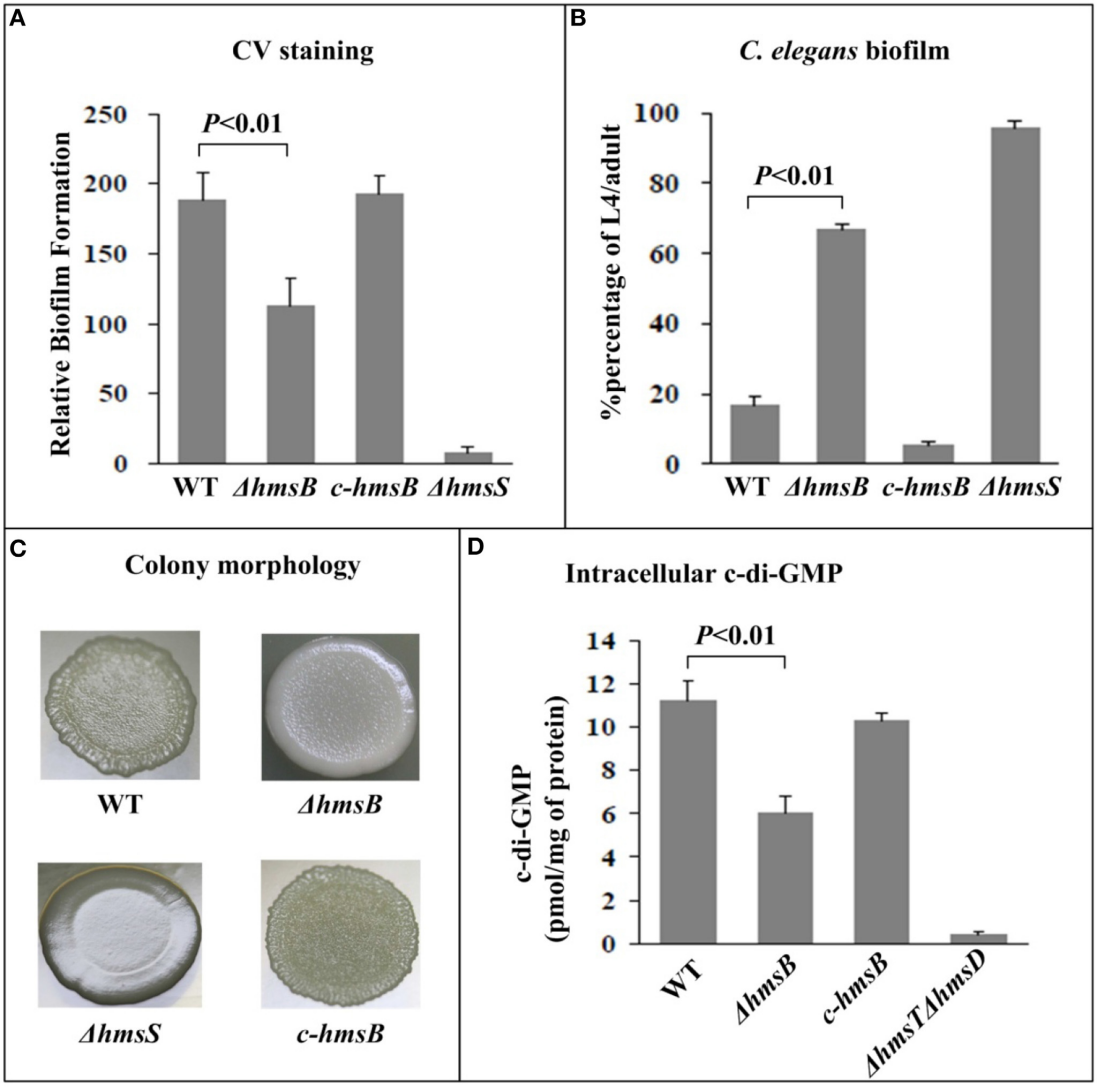
**Involvement of HmsB in biofilm formation and c-di-GMP biosynthesis. (A)** Crystal violet staining. *Y. pestis* was grown in 24-well polystyrene dishes, and the bacterial biomass (*in vitro* biofilms) attached to well walls were stained with crystal violet to determine OD_570_ values. The planktonic cells were subjective to determine OD_620_ values. The relative capacity of biofilm formation of each strain tested was shown with values of 500 × OD_570_/OD_620_. **(B)** *C. elegans* biofilms. After incubation of nematode eggs on lawns of indicated *Y. pestis* strains, the developmental stages of nematodes on each lawn were scored to calculate percentage of L4/adult. (**C)** Bacterial colony morphology. Aliquots of bacterial glycerol stocks were spotted on LB plate, followed by incubation for one week. (**D)** Intracellular c-di-GMP concentration. The intracellular c-di-GMP concentrations were determined by a HPLC-MS/MS method, and the determining values were expressed as pmol/mg of bacterial protein.

Determination of the growth curves of WT, Δ*hmsB and c-hmsB* showed that the *hmsB* deletion had no affect on bacterial growth *in vitro* (data not shown). Crystal violet could steadily stain *in vitro* biofilm masses produced by WT or *c-hmsB*; by contrast, ΔhmsB stained a great deal less crystal violet (Figure 2A), and as expected, almost no crystal violet straining could be detected for the reference biofilm-negative strain ΔhmsS. After incubation of nematode eggs on bacterial lawns of WT or *c-hmsB*, only a small portion (below 20%) of larvae grew and developed to L4/adult nematodes due to abundant attachment of *Y. pestis* biofilms on nematode heads; by contrast, bacterial lawns of ΔhmsB and ΔhmsS gave the percentage values of about 65 and 100%, respectively (Figure 2B). These indicated that the *hmsB* deletion compromised biofilm formation both *in vitro* and on nematodes. When grow on agar plates, WT and *c-hmsB* gave similar rugose colony morphology due to abundant biosynthesis of exopolysaccharide, Δ*hmsS* produced very smooth colonies, while ΔhmsB lied between ΔhmsS and WT/*c-hmsB* (Figure 2C). Intracellular c-di-GMP concentrations were determined in WT, ΔhmsB, and *c-hmsB* by a HPL-MC/MS method. Compared to WT or *c-hmsB*, a significantly decreased production of c-di-GMP was observed for ΔhmsB (Figure 2D). As expected, almost no c-di-GMP could be detected for the reference c-di-GMP-negative strain ΔhmsT ΔhmsD. In addition, there were similar observations of bacterial growth curve, c-di-GMP concentration, and crystal violet staining of biofilms in WT, ΔhmsB and *c-hmsB* (data not shown), when bacteria were grown in Brain Heart Infusion (BHI) broth or in chemically defined TMH medium (Straley and Bowmer, 1986). Taken together, the above results indicated that HmsB enhances c-di-GMP and exopolysaccharide production, which could account for HmsB-dependent lesion of biofilm formation.

*hmsT*, *hmsHFRS*, *hmsCDE*, *hmsP* and its own gene were subjected to the following gene regulation assays for characterization of HmsB-dependent expression of these target genes. Levels of gene expression and protein biosynthesis were determined in WT and ΔhmsB but not the complemented mutant strain *c-hmsB*. This design was based on the following two observations: *c-hmsB* and WT gave very similar c-di-GMP and biofilm phenotypes (see above); and no change in expression of *hmsC* (upstream of *hmsB*) or *mgtA* (downstream) was detected in *c-hmsB* relative to WT by using quantitative RT-PCR and primer extension (data not shown).

**Figure 3:**
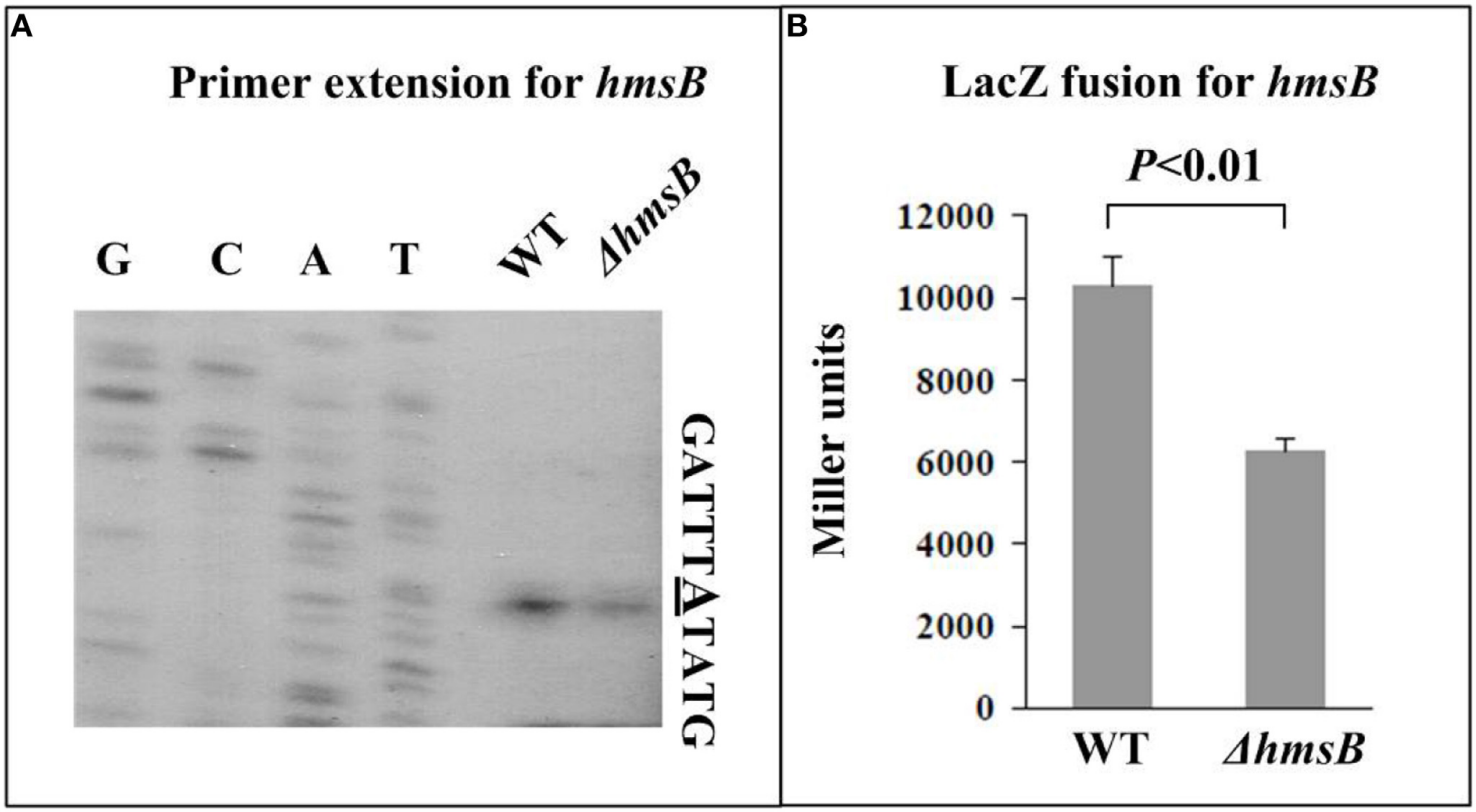
**HmsB-dependent expression of *hmsB*. (A)** Primer extension. The mRNA levels of *hmsB* in WT or ΔhmsB were determined by primer extension. The Sanger sequence ladders (lanes G, C, A, and T) and the primer extension products of *hmsB* were analyzed with an 8 M urea-6% acrylamide sequencing gel. The transcription start site of *hmsB* was indicated by underlined nucleotide A. **(B)** LacZ fusion. The P*hmsB*:*lacZ* transcriptional fusion vector was transformed into WT or ΔhmsB, and then the *hmsB* promoter activities (miller units of β-galactosidase activity) were determined in bacterial cellular extracts.

**Figure 4:**
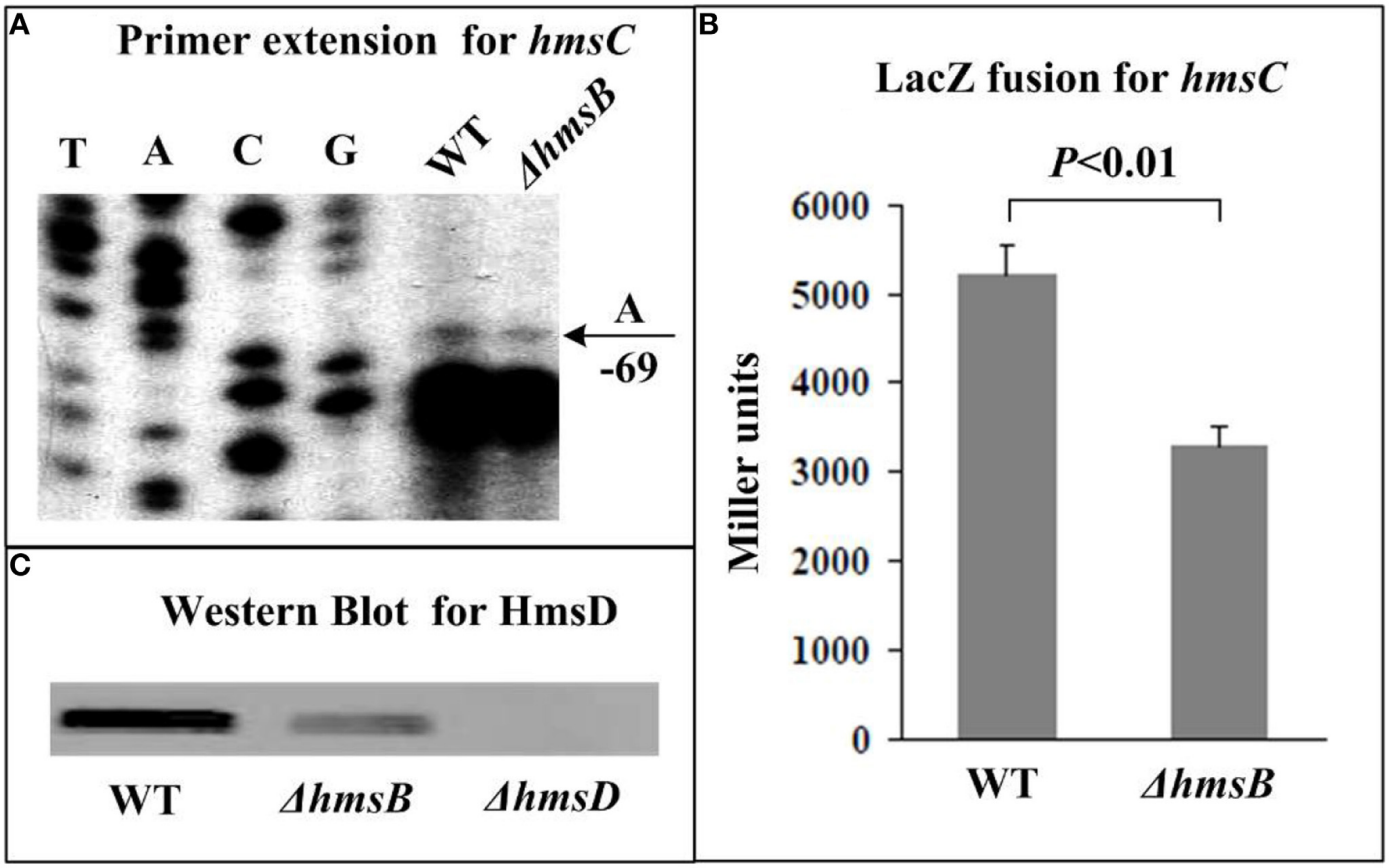
**HmsB-dependent expression of *hmsCDE***. Primer extension **(A)** and LacZ fusion **(B)** experiments were done for *hmsC* as described in Figure 3. The 5′ terminus of RNA transcript (i.e., transcription start) of *hmsC* was indicated by arrow with nucleotide A, and the minus numbers under arrow indicated nucleotide position upstream of *hmsC*. For Western Blot **(C)**, whole-cell protein extract from WT or ΔhmsB or ΔhmsD (negative control) was loaded for SDS-PAGE and incubated with anti-HmsD antibodies. Noted that the first gene of *hmsCED* was subjected to gene expression assays **(A,B)**, while diguanylate cyclase HmsD was chosen for protein biosynthesis analysis **(C)**.

**Figure 5:**
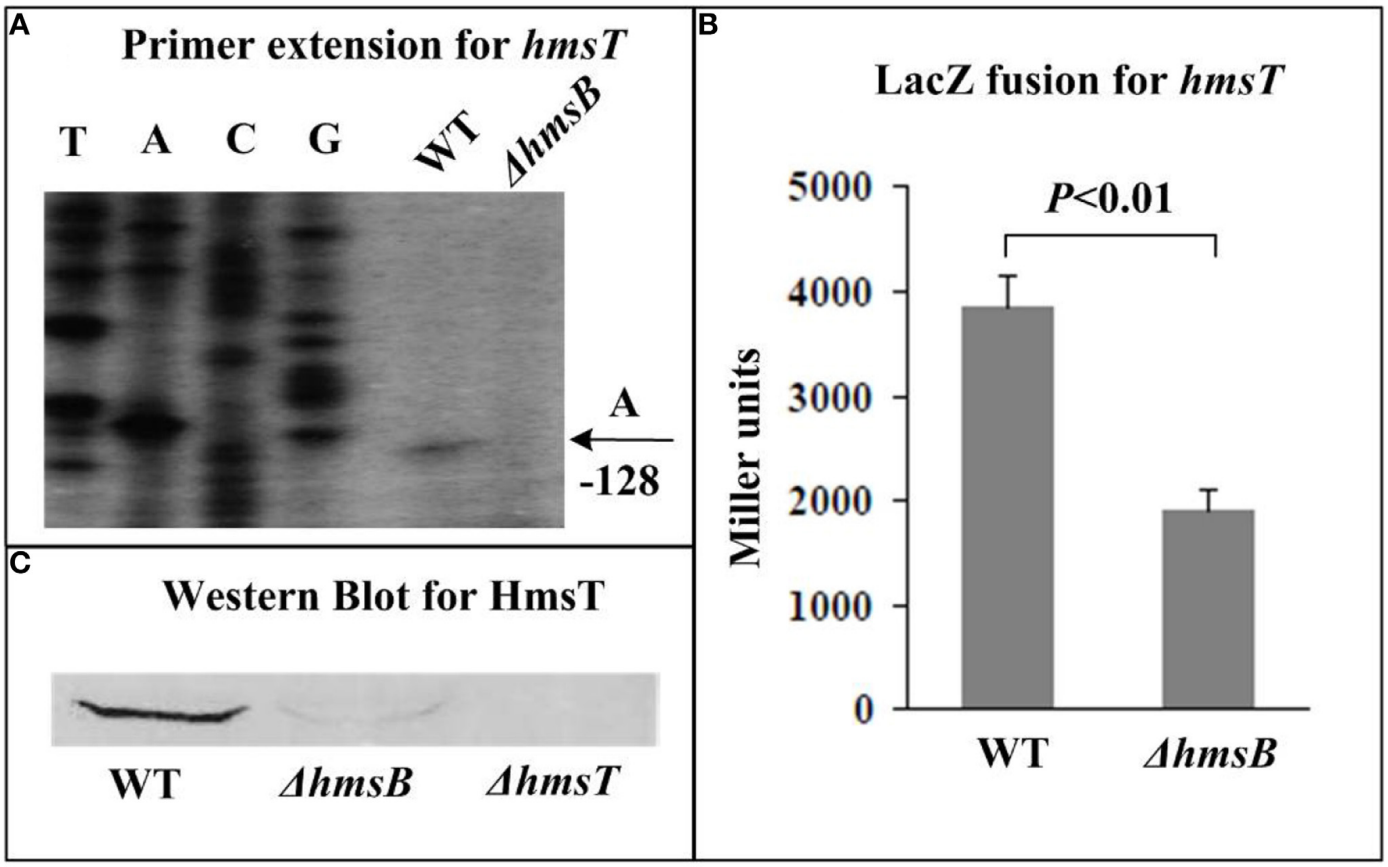
**HmsB-dependent expression of *hmsT***. Primer extension **(A)** and LacZ fusion **(B)** for *hmsT*, and Western Blot **(C)** for HmsT were done as described in Figure 3.

**Figure 6:**
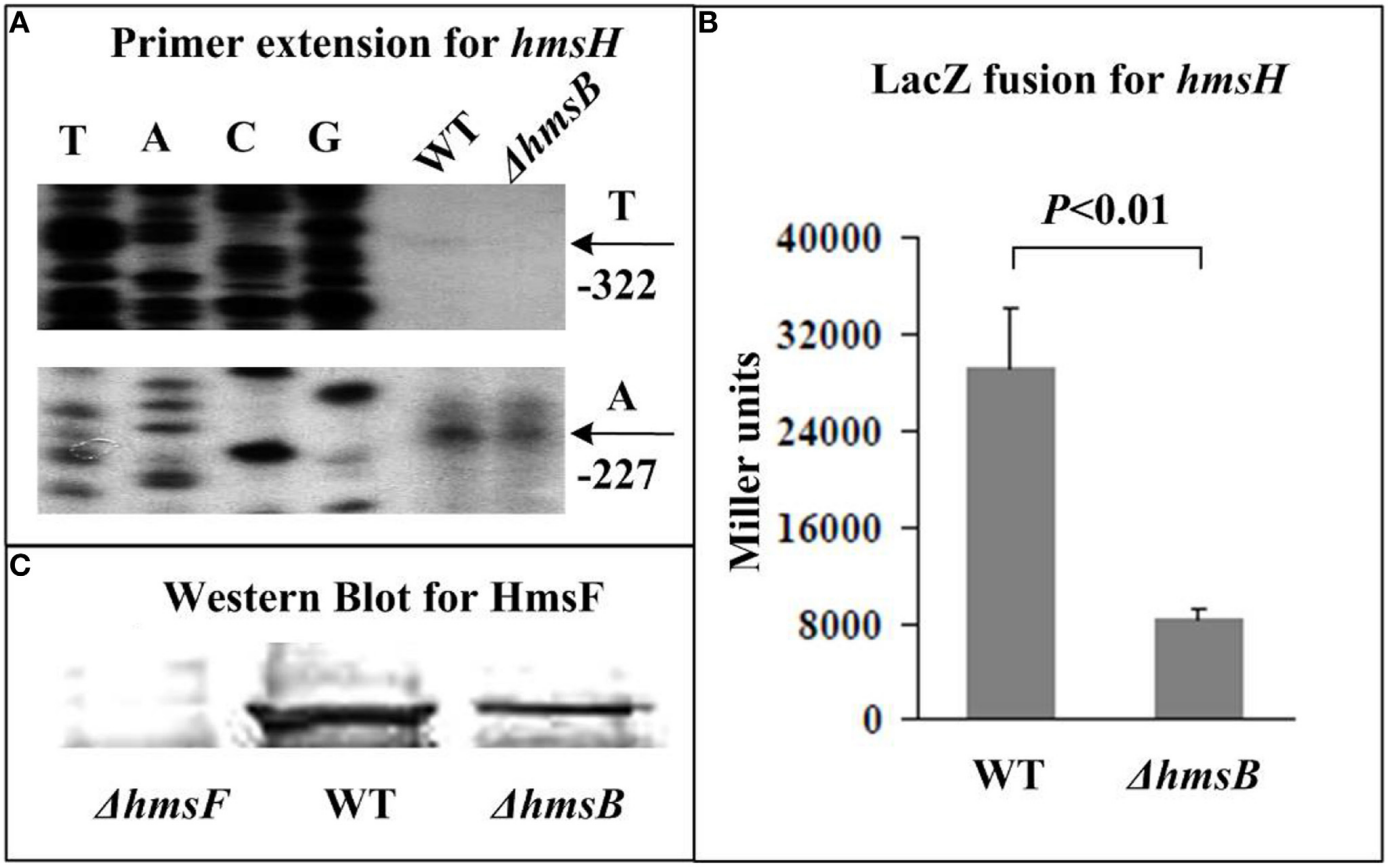
**HmsB-dependent expression of *hmsHFRS***. Primer extension **(A)** and LacZ fusion **(B)** for *hmsH*, and Western Blot **(C)** for HmsF were done as described in Figure 3. Noted that the first gene of *hmsHFRS* was subjected to gene expression assays **(A,B)**, while polysaccharide deacetylase HmsF was chosen for protein biosynthesis analysis **(C)**.

The relative mRNA level of each of *hmsB* (Figure 3A), *hmsC*(Figure 4A), *hmsT* (Figure 5A), and *hmsH* (Figure 6A) was measured in WT or ΔhmsB by primer extension assay, and the results showed that the mRNA level of each of the above four genes decreased considerably in ΔhmsB relative to WT. Notably, this assay detected a single transcription start site (nucleotide A) located at nucleotide position 472430 on CO92 genome, which confirmed the above 5′-RACE result. The promoter-proximal region of each of *hmsB* (Figure 3B), *hmsC* (Figure 4B), *hmsT* (Figure 5B), and *hmsH* (Figure 6B) was cloned into the transcriptional *lacZ* fusion reporter vector pRW50, and the corresponding recombinant vector was introduced into WT or ΔhmsB to determine the target promoter activity; it was shown that the promoter activity of each of the above four genes was significantly reduced in ΔhmsB relative to WT. Further Western blot assay confirmed that biosynthesis of each of HmsD (Figure 4C), HmsT (Figure 5C), and HmsF (Figure 6C) decreased in ΔhmsB relative to WT. Notably, observations from transcriptional *lacZ* fusion experiments denoted that HmsB-dependent expression of *hmsB*, *hmsCDE*, *hmsT*, and *hmsHFRS* most likely involved mechanisms of gene transcriptional regulation.

**Figure 7:**
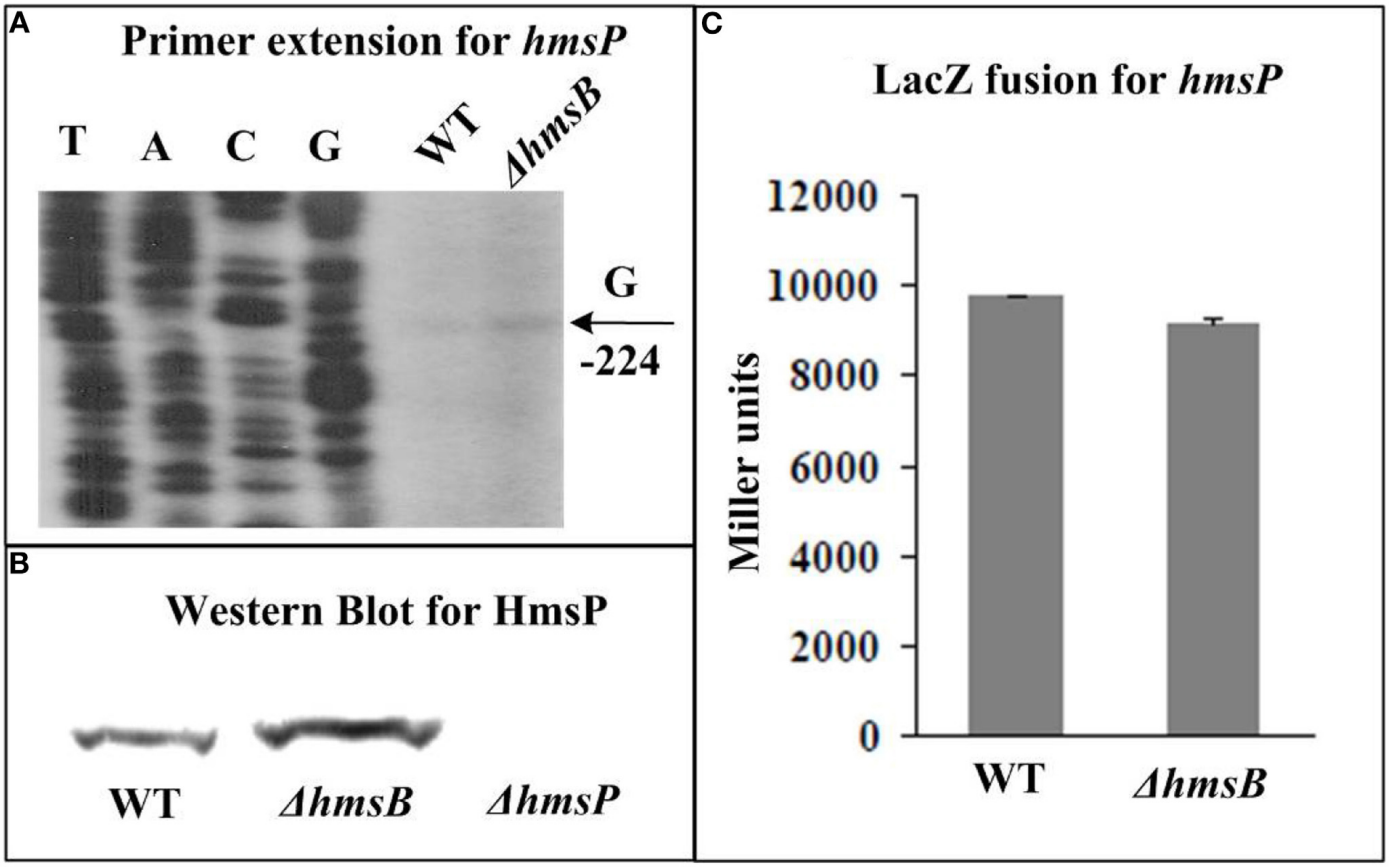
**HmsB-dependent expression of *hmsP***. Primer extension **(A)** and LacZ fusion **(C)** for *hmsH*, and Western Blot **(B)** for HmsP were done as described in Figure 3. Noted that each of *hmsB* (Figure 3), *hmsCDE* (Figure 4), *hmsT* (Figure 5), and *hmsP* (this figure) had a single transcription start site, while *hmsHFRS* (Figure 6) had two different ones (see our companion submission for details).

By contrast, primer extension (Figure 7A) and Western blot (Figure 7B) assays indicated negative regulation of *hmsP* by HmsB at mRNA and protein levels, respectively. Further transcriptional *lacZ* fusion experiments (Figure 7C) indicated that HmsB had no regulatory effect on *hmsP* promoter activity.

## Discussion

Data presented here showed that *Y. pestis* sRNA HmsB enhanced the production of c-di-GMP, exopolysaccharide, and biofilm. In addition, HmsB stimulated expression of *hmsB*, *hmsCDE*, *hmsT*, and *hmsHFRS*, all of which encoded biofilm-enhancing factors, while repressed that of *hmsP* encoding a biofilm-inhibiting factor. HmsB appeared to act as a major activator of biofilm formation in *Y. pestis*. To the best of our knowledge, this is the first report of a sRNA regulating *Yersinia* biofilm formation.

HmsB had regulatory effect on promoter activity of *hmsB*, *hmsCDE*, *hmsT*, and *hmsHFRS* but not that of *hmsP*. Commonly, sRNAs inhibit the translation of their mRNA targets by base paring with the neighborhoods of ribosomal binding sites (RBSs) to block ribosome binding and thus to inhibit protein biosynthesis (Han et al., 2013). Less commonly in cases studied to date, sRNAs can activate translation by freeing RBSs that would otherwise be occluded by inhibitory secondary structures (Han et al., 2013). Whether HmsB binds to RBS-around regions of these *hms* genes needs to be elucidated.

The positive regulatory action of HmsB on the promoter activity of *hmsB*, *hmsCDE*, *hmsT*, and *hmsHFRS*, as characterized in this work, are highly unusual; it is speculated that HmsB modulates the translation of one or more transcriptional activators or repressors of the above *hms* genes. It should be noted that multiple transcriptional regulators of *Y. pestis* biofilm formation have been identified (Sun et al., 2012; Rebeil et al., 2013; Tam et al., 2014).

The *hfq* deletion led to dramatic degeneration of HmsB in *Y. pestis* (unpublished data). Most of sRNAs characterized to date need binding of Hfq as a RNA chaperone, stabilizing formation of imperfect sRNA-target RNA duplexes (Han et al., 2013). It has been characterized that Hfq is essential for the biofilm formation and flea blockage of *Y. pestis* strain KIM6+ during colonization of flea gut (Rempe et al., 2012). Positive control of biofilm formation by Hfq is also observed in *Y. pestis* strain 201 used in this study, and further gene regulation assays show that Hfq enhancs the expression of *hmsCDE*, *hmsT*, and *hmsHFRS* but inhibits that of *hmsP* in this strain (unpublished data). By contrast, a separate study reports that Hfq is a repressor of biofilm formation through inhibiting expression of *hmsCDE*, *hmsT*, and *hmsHFRS* but stimulating that of *hmsP* in a pCD1- derivate of *Y. pestis* CO92 (Bellows et al., 2012); interestingly, similar results can be observed in *Y. pestis* strain 201 cured of pCD1 (unpublished data).

The sRNA Ysr141 targets an untranslated region upstream of *yopJ* to posttranscriptionally activate synthesis of YopJ, an effector protein of the Yop-Ysc type III secretion system (Schiano et al., 2014); this is up to now the only report of sRNA-target gene association in *Y. pestis*. Although HmsB-dependent expression *hms* genes have been dissected in the present work, direct HmsB targets (probably including not only *hms* genes but their upstream transcriptional regulators) as well as detailed mechanisms of action of HmsB in aid of Hfq on its target genes needs to be dissected to understand how HmsB contributes to biofilm gene regulation.

### Conflict of interest statement

The Associate Editor, Yicheng Sun, declares that, despite having collaborated with author, Dongsheng Zhou, on the same Research Topic, the review process was handled objectively. The authors declare that the research was conducted in the absence of any commercial or financial relationships that could be construed as a potential conflict of interest.
