# Supplementary material for: HmsB enhances biofilm formation in Yersinia pestis
Source: Front Microbiol. 2014 Dec 12;5:685. doi: 10.3389/fmicb.2014.00685 (PMC4264472; doi:10.3389/fmicb.2014.00685)
Supplement: Supplementary file 1 [file Table1.DOC]

**Table S1 Primer used in this study**

| **Target**  **gene** | **Primers (5'-3'; F/R)** |
| --- | --- |
| **Mutant construction** | |
| *hmsB* | TTATCTTAAATCTTATTTATGTTATTGAAATAGAAATATAAAAAACCCGACTGTTTTCCGAGATTGCAGCATTACACG/GTCAGAAGAGAAAAAGCCCCGGAGCAGTCCAGCCCAGCTCCGGGGCTTTTGAGGTAAATATGTAACGCACTGAGAAGC |
| *hmsT* | AAATACCTCACGAGGATATGATTTATTAGTCTACTGACAGCACGA AGATTGCAGCATTACACG/ATCATTAAAAATAATAACTGGCATAGAGCGTCCGTATGTTCAGTG TGTAACGCACTGAGAAGC |
| *hmsD* | ATGAAGCCGAAAAAAAATCACAATAGCGCAGAAAAACTCCAGATTGCAGCATTACACGTC/CTATCCTAAACTTTCTGTGTTAGTCGTATTCGGCTGATAGTGTAACGCACTGAGAAGC |
| *hmsP* | CCACCTTATTTTATGCAGTCGGAATTGGCGGAGAACGAGTAGATTGCAGCATTACACGTC/GCGTGTTAAGTACTGCTGGTGGACACCGCCATACAAAGAGTGTAACGCACTGAGAAGC |
| *hmsF* | ACGATTCAGCCGATCAACGCTATCTGTCGGTAAGAAGCAGAGATTGCAGCATTACACGTC/CCAAAGCACCGGGGCGTTGCGCAACTTTATTGACTAGCTCTGTAACGCACTGAGAAGC |
| **Complementation of the mutant** | |
| *hmsB* | ACCGTCGACCAGTGCTGGATTAAATAACATTTC/ GAAGGATCCGACCTTGATTGCTTGTAGTG |
| **Protein expression** | |
| *hmsT* | GCGG***GGATCC***GCTGTGCGTTCAAGAGAT/ GCGG***GTCGAC***TCAAGGGGAAGACTGTAC |
| *hmsF* | GCGG***GAATTC***ATTGCTAACCCACAGGGGAATA/ GCGG***AAGCTT***CTACCGGATAGCATCGG |
| *hmsD* | GCGG***GGATCC***GAGCTCAGTAATGACTTT/ GCGG***GTCGAC***CTATCCTAAACTTTCTGT |
| *hmsP* | GCGG***GGATCC***CGCCAGCAGGTTATCGGTGC/ GCGG***GTCGAC***TTAACTTACGTGGTGAGCGCTG |
| **Primer extension** | |
| *hmsH* | /TATTGTTGCAAAGTCATTATAGGAT |
| *hmsT* | /GGTATTTATTCCGACATCACGAC |
| *hmsC* | /AGTAGCGGTAGTCATTTTTACG |
| *hmsP* | /CCATCGAGTAAGTTGTGATCC |
| *hmsB* | /CAGTCGGGTTTTTTATATTTC |
| **LacZ reporter fusion** | |
| *hmsH* | GCGGGATCCACTTTGCTGAAGACTTGTCACG/ GCGAAGCTTCCGCCATAGCAGGATTAACG |
| *hmsT* | GCGGAATTCGCCCAGTACAGGTAACAAGG/ GCGGGATCCCTGATCGTAGGAGTGGCTATTC |
| *hmsC* | TCTGGATCCCTTACTGGTTGCTATTGCC/ TCTAAGCTTGAGGTTCATGATGTTCATCA |
| *hmsP* | GCGGGATCCAGCGATGGTAGAAGTGAATCAG/ GCGAAGCTTTTGCGATACTCTAATGGAAGGC |
| *hmsB* | TCTGGATCCGTAAGAATAGATAAACGCCCCACC/ TCTAAGCTTCTACTTCCCCTACTCGTGCTTCAG |
| ***5’-RACE*** | |
| *hmsB* | TCAACTACCTGACAAAAACCCG/TCGGGTTTTTTATATTTCTATTTC |
| ***3’-RACE*** | |
| *hmsB* | TTTTCCGGGTTTTTGTCAGGTAG/AAGCACGAGTAGGGGAAGTAGACC |
